# Quantum-enhanced time-domain spectroscopy

**DOI:** 10.1126/sciadv.adt2187

**Published:** 2025-01-24

**Authors:** Dionysis Adamou, Lennart Hirsch, Taylor Shields, Seungjin Yoon, Adetunmise C. Dada, Jonathan M. R. Weaver, Daniele Faccio, Marco Peccianti, Lucia Caspani, Matteo Clerici

**Affiliations:** ^1^ James Watt School of Engineering, University of Glasgow, Glasgow G12 8QQ, UK.; ^2^ School of Physics and Astronomy, University of Glasgow, Glasgow G12 8QQ, UK.; ^3^ Emergent Photonics Research Centre, Department of Physics, Loughborough University, Loughborough LE11 3TU, UK.; ^4^ Institute of Photonics, Department of Physics, University of Strathclyde, Glasgow G1 1RD, UK.; ^5^ Como Lake Institute of Photonics, Dipartimento di Scienza e Alta Tecnologia, Università degli Studi dell’Insubria, Via Valleggio 11, 22100 Como, Italy.

## Abstract

The time-resolved detection of mid- to far-infrared electric fields absorbed and emitted by molecules is among the most sensitive spectroscopic approaches and has the potential to transform sensing in fields such as security screening, quality control, and medical diagnostics. However, the sensitivity of the standard detection approach, which relies on encoding the far-infrared electric field into amplitude modulation of a visible or near-infrared probe laser pulse, is limited by the shot noise of the latter. This constraint cannot be overcome without using a quantum resource. Here, we show that this constraint can be overcome using a two-mode squeezed state. Quantum-correlated ultrashort pulses, generated by parametric down-conversion, enhance the sensitivity of far-infrared detection beyond the classical limit, achieving a twofold reduction in measured noise. This advancement paves the way for further development of ultrafast quantum metrology, moving toward quantum-enhanced time-resolved electric field spectroscopy with sensitivities beyond the standard quantum limit.

## Teaser

Two-mode squeezed states allow for a twofold noise reduction in time-resolved electric field sensing.

## INTRODUCTION

Optical spectroscopy is a powerful technique that underpins fundamental research and applications alike. It can be used to measure the chemical composition of a tested sample; to assess the safety of foods, air, or water; and to reveal the interaction of molecules in a complex living system (*1*–*7*). With the continuous development of ultrashort laser sources, an alternative paradigm for spectroscopy has developed, whereby spectral signatures are obtained by the Fourier transform of the electric field of a probing laser pulse (*8*–*12*). This time-resolved electric field spectroscopy (from here on, time-domain spectroscopy—TDS—for brevity) provides additional information unavailable to standard spectroscopic approaches, such as time-of-flight longitudinal localization of chemical species, a higher sensitivity to the interaction between the environment and the investigated molecule, and an unparalleled sensitivity in living samples (*12*). TDS was first pioneered in the terahertz spectral region (THz-TDS), where femtosecond pulses are sufficiently short to properly sample and resolve the temporal oscillations of the electric field of a carrier-envelope–phase stable and broadband (single-cycle) pulse generated by the optical rectification (OR) of a short optical pump pulse. With the development of ultrafast sources, it became possible to extend the spectral domain of TDS to cover the whole infrared region, reaching even to visible wavelengths (*13*–*15*). The ability to sample such a broadband spectrum is key to the unparalleled specificity of TDS (*12*).

The measurement process is routinely achieved by electro-optical sampling (EOS), whereby the unknown electric field is transformed by a second-order nonlinear (electro-optical) crystal into a phase shift of the short optical probe pulse, which is then measured by a balanced detector (*16*, *17*). While research is ongoing to establish the maximum efficiency of EOS (*18*), its sensitivity is ultimately limited by the noise of the probe pulse, which is, in turn, bound by its discretization (shot) noise (*19*, *20*). The increase in signal-to-noise ratio (SNR) obtained by increasing the probe pulse energy is proportional to the square root of the photon number, $\text{SNR}\propto \sqrt{N}$, and cannot be made arbitrarily large due to nonlinear noise and back action (*21*). Therefore, it is only by using nonclassical states of light, where Heisenberg-limited SNR ∝ *N* can be achieved (*22*–*25*), that we can overcome the current limitations faced by EOS (*26*).

Here, we show experimental results of a quantum-enhanced TDS. Using two-mode squeezed states, i.e., quantum-correlated fields generated by parametric down-conversion in a second-order nonlinear crystal (*27*), we were able to record a THz electric field with a noise of half the standard quantum limit.

## RESULTS

### THz generation

**Fig. 1.**
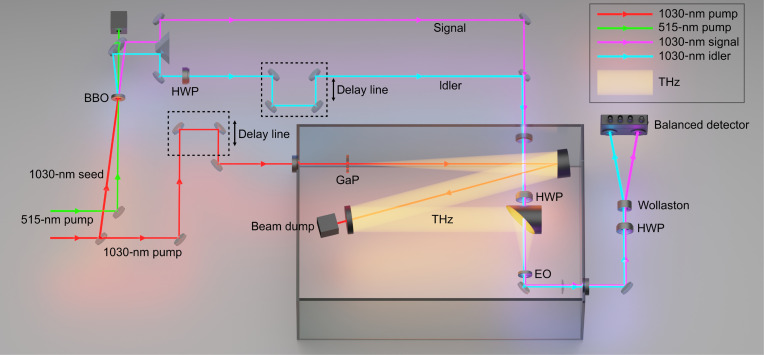
Schematic of the experimental setup. A 1030-nm, 250-fs-duration, 100-kHz-repetition-rate laser is split into two branches. The bottom branch (in red in the figure) pumps a GaP crystal, leading to the generation of a single-cycle THz pulse (yellow). The top branch (p-polarized, also in red) seeds a parametric amplifier pumped by a synchronized laser (s-polarized, green in the figure) at 515 nm, generating a two-mode squeezed vacuum (p-polarized), consisting of photon number correlated signal (purple) and idler (cyan) pulses. The idler polarization is rotated to s-polarization using a half-wave plate (HWP). The signal pulse is used for the electro-optical (EO) detection of the THz electric field while the idler is delayed to not interact with the THz pulse and serves as a reference. The EO modulation is analyzed by a polarimetric arrangement comprising an HWP, a Wollaston polarizer, and a low-noise, high–quantum-efficiency balanced detector. The temporal resolution is achieved by delaying the THz with respect to the signal and idler pulses using a linear translation stage. Note that the idler polarization impinging on the EO crystal is orthogonal (s-polarized) to that of the signal, and both are rotated by 45° with an HWP before interacting with the linearly polarized THz field in the EO crystal, and then are rotated back to almost the same initial condition before reaching the Wollaston prism.

In our experiment, we generate trains of single-cycle THz radiation via OR of a 250-fs-duration, 1030-nm-wavelength, 100-kHz-repetition-rate pulse train in a 1-mm-thick, (110)-cut, antireflection coated, water-cooled gallium phosphide (GaP) crystal, owing to the favorable matching between the driving pulse group velocity and the THz radiation phase velocity (*28*). Using 45 μJ energy for the pump, we generated 150 nJ of THz radiation after suitable filtering of the residual pump light. A schematic of the experimental setup is shown in Fig. 1. The region where THz radiation propagates is enclosed in a nitrogen-purged box to avoid water absorption. More details of the THz source can be found in (*29*).

### Electro-optical sampling

The time-resolved electric field detection of the THz radiation is performed by exploiting the electro-optical effect in a second-order nonlinear crystal between the unknown THz field ${ℇ}_{\text{THz}}$ and a short probe pulse of intensity $I_{p}$. When the probe pulse duration is much shorter than half of the period of the oscillations in ${ℇ}_{\text{THz}}$, the latter can be considered as a static field that biases the nonlinear crystal, thus imposing a field-dependent phase upon the probe pulse. Such a phase, and therefore the THz electric field, can be measured by a polarimetric setup typically consisting of a quarter-wave plate (QWP), a polarizing beam splitter (PBS; e.g., a Wollaston prism), and a balanced detector (*17*, *30*, *31*). In the absence of the THz field, the two output ports of the PBS have equal intensities, while the THz-induced phase leads to an imbalance that is transformed into an electric signal by the balanced detector and captured by a lock-in amplifier (the amplitude of the THz field is modulated, e.g., by an optical chopper, at the lock-in demodulation frequency). The demodulated signal is then proportional to the THz electric field ${ℇ}_{\text{THz}}\left(\tau \right)$ at the local time τ sampled by the probe pulse. The temporal information is retrieved varying the relative delay τ between the THz and the probe pulses. In our case, we used a modified version of the standard EOS (see the “THz generation” and “Electro-optical sampling” sections in Materials and Methods), which uses a half-wave plate (HWP) in place of the QWP (see Fig. 1), as it leads to a simpler demonstration of the enhancement that can be achieved by using quantum metrology tools, albeit featuring a lower sensitivity. An insightful analysis of different EOS settings can be found in (*30*), demonstrating, for example, how EOS performed with an HWP, combined with spectral filtering of the low- or high-frequency components of the upconverted radiation, results in the Hilbert transform of the THz field. In our demonstration, we follow a different approach that can be regarded as relative ellipsometry (using an HWP) to retrieve a signal proportional to the THz electric field, and the imbalance in the intensities measured by the balanced detector is proportional to the THz electric field ${ℇ}_{\text{THz}}(\tau )$.

Among other noise sources, such as amplitude noise of the unknown field, limited accuracy of the scanning optical delays, and the timing jitter of the probe and THz pulses, the probe pulse shot noise in the balanced detection makes a leading contribution to limiting the sensitivity of EOS (*19*, *20*). Considering a single probe pulse of energy $U_{p}$, a balanced measurement where the energy is equally split between the two diodes is affected by a white noise of amplitude proportional to $\sqrt{N_{p}}$, where $N_{p}=U_{p}/\left(\mathrm{h\nu}\right)$ is the number of photons in the probe pulse, with h being the Planck constant and ν being the probe carrier frequency. We show here that this inherent noise source can be largely reduced by using, instead of a coherent pulse, two-mode squeezed states, i.e., quantum correlated fields, also known as twin beams. To this end, we have compared the EOS noise measured with a classical probe to that obtained using two-mode squeezed states, and we have shown that the latter is markedly lower.

### Two-mode squeezed states

In a parametric amplification process driven by an intense laser pulse in a second-order nonlinear crystal, a weak seed is amplified by the stimulated splitting of the pump photons into signal and idler photons (*32*). These are always generated in pairs at each pump photon splitting event. Hence, their numbers $N_{s}$ and $N_{i}$ are correlated, and the variance of $N_{s}-N_{i}$ is lower than that which can be achieved with classical radiation of an equivalent power on each detector (*27*):

$$\Delta^{2}{\left(N_{s}-N_{i}\right)}_{\text{PDC}}<\Delta^{2}{\left(N_{s}-N_{i}\right)}_{\text{Classical}}$$ (1)

where the variance of an observable *O* is defined as $\Delta^{2}\left(O\right)\equiv \langle O^{2}\rangle -{\langle O\rangle }^{2}$, 〈·〉 indicates the expectation value, and $\Delta^{2}{\left(N_{s}-N_{i}\right)}_{\text{Classical}}=\langle N_{s}\rangle +\langle N_{i}\rangle$ is the variance in the number difference between the two output ports of an ideal 50/50 beam splitter having at the input a coherent beam of average photon number $\langle N_{c}\rangle =\langle N_{s}\rangle +\langle N_{i}\rangle$. Therefore, in differential measurements, two-mode squeezed states can deliver a quantum advantage that has been applied, e.g., to imaging (*33*, *34*), spectroscopy (*35*), and differential absorption sensing (*36*). Here, we use this quantum resource to increase the SNR of our EOS process.

**Fig. 2.**
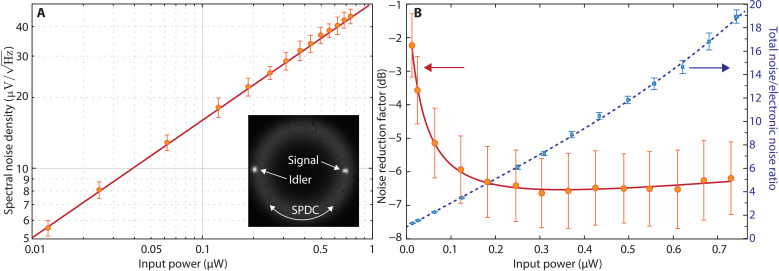
Noise analysis. (**A**) Spectral noise density of the differential signal recorded by our balanced detector illuminated by two coherent sources of equal amplitude as a function of the total power impinging on the two photodiodes (input power). The red line is the expected shot noise. The experimental data match well with the theoretical prediction, confirming that the detection is shot-noise limited. The inset shows a far-field image of the radiation generated by parametric down-conversion in the BBO crystal. The faint ring is the spontaneous PDC (SPDC) radiation while the two spots are the amplified seed (signal) and the idler beams. (**B**) Left axis: noise reduction factor (NRF) in the decibel scale for the squeezed source and detector used in our experiment and recorded using the setup shown in Fig. 1 in the absence of the THz signal. Note that the NRF is computed without subtraction of the electronic noise. The red curve is a fit of the data that serves as a guide for the eye. Right axis: ratio of the total measured noise to the electronic noise.

To exploit sub shot-noise correlations, photodetectors with appropriate gain and low noise are necessary, such that the electronic noise is smaller than the optical shot-noise component. We have designed and built a balanced detector with the required electrical noise (50 fA Hz^−1/2^) and transimpedance gain (10^6^ ohms) and with high (>94% external) quantum efficiency—see the “Sub shot-noise balanced detector” section in Materials and Methods for details. Our detector can measure the optical shot noise for coherent pulses over a broad range of average power, including those used in our nonclassical measurement, as shown in Fig. 2A, where the error bars also account for the electronic noise. The detection was performed with a power spectrum analyzer acquiring 100 traces at each input power in a spectral region between 2.1 and 2.9 kHz (the same region sampled by the lock-in amplifier in the following THz time-resolved measurements) and averaging the traces to compute the spectral noise density.

Our two-mode squeezed source was obtained by pumping a 2-mm-long beta barium borate (BBO) crystal with the 1.5-W, 515-nm-wavelength, ≃185-fs-duration second harmonic pulse of our Yb-doped laser (Carbide, Light Conversion) operating at 100-kHz repetition rate. The crystal was angle tuned to amplify frequency degenerate radiation at ~1030 nm in a slightly noncollinear configuration, i.e., on a ~2° cone angle.

While the spontaneously generated parametric down-conversion (PDC) radiation resulting from the parametric amplification of vacuum fluctuations also features quantum correlations among the number of photons in modes emitted at opposite angles from the crystal, we used a seeded geometry. In this way, the spatiotemporal modes resulting from the amplification are defined by the input seed and the pump spatial and temporal properties. This allows for the required control of the spatiotemporal overlap between the probing pulse and the unknown THz field.

The parametric amplification was seeded with a 1030-nm pulse of ~245 fs duration, impinging on the crystal at an angle of ≃2° from the pump pulse (see the “Two-mode squeezed light source” section in Materials and Methods). This angle corresponds to the noncollinear emission angle for degenerate PDC and resulted in the generation of the correlated signal and idler beams shown in the inset of Fig. 2A. Provided the parametric amplification gain is sufficiently large, and the seed amplitude is sufficiently low, the seeded geometry also results in a quantum correlated two-mode squeezed field (*37*–*40*). The quality of the correlation between the number of photons in the signal and the idler can be quantified by the noise reduction factor (NRF), defined as in (*27*)

$$\text{NRF}=\frac{\Delta^{2}\left(N_{s}-N_{i}\right)}{N_{s}+N_{i}}$$ (2)

The quantum correlations are reduced by losses (*41*), which limit the NRF. We have used broadband antireflection coatings on all the transmissive optics in the setup and high reflectivity coatings on the reflective ones to minimize the impact of losses in our measurement. For the same reason, we built a balanced detector with photodiodes having external quantum efficiencies larger than 95% (see the “Sub shot-noise balanced detector” section in Materials and Methods). We have estimated <15% losses from generation to detection. The best NRF measured for our source passing through all the optical elements required for the EOS is shown in Fig. 2B (left axis). Such a measurement has been obtained at a constant pump power of 1.5 W while varying the seed power between 0.5 and 25 nW.

Considering a probe power in the 0.1 to 1 μW range for our quantum-enhanced EOS, from the recorded NRF, we expect at least a twofold decrease in the measured THz electric field noise. We note that the NRF increases at low probe power due to the increasing contribution of the detector electronic noise to the overall noise. This is evidenced by observing the ratio between the overall noise and the electronic noise shown in Fig. 2B (right axis). At low signal powers, the electronic noise is the dominant component. We also note that the NRF slowly increases at high two-mode squeezed pulse powers due to the limited common mode rejection ability of the detector.

### Sub shot-noise EOS

**Fig. 3.**
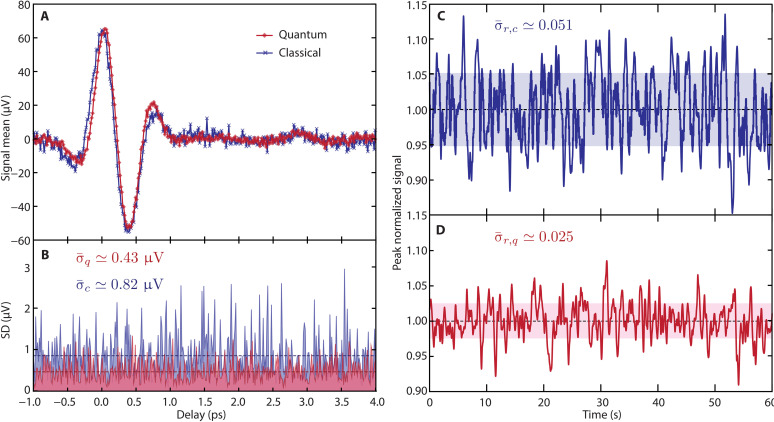
Noise analysis for the THz-TDS measurement. (**A**) Time-resolved THz electric field amplitude measured with the approach described in the text. The error bars on both curves (classical in blue and quantum enhanced in red) are obtained from the experimental data calculating the SD of the spectral noise density within a selected frequency band. (**B**) Standard deviation of the electric field resolved in time for the classical (blue) and quantum (red) acquisitions. The dashed lines are the averages in both cases (with values reported in the figure panel). (**C**) and (**D**) are the normalized electric field value acquired over 1 min for the classical and quantum measurements, respectively. Values within 1 SD are shown in both cases with a shaded area.

Using the two-mode squeezed source discussed above, we measured the THz electric field with our EOS scheme, and we compared the result with what can be achieved in the same scheme using a standard classical probe, i.e., a coherent pulse from the laser while maintaining the same average number of photons per pulse on the balanced detector. The results are shown in Fig. 3. In Fig. 3A, we report the result of a time-resolved measurement of the THz electric field using either the two-mode squeezed beam (red) or the coherent (blue) probe pulse of the same power (≃0.6 μW). The noise in the quantum case is smaller than in the classical one. As both measurements are performed under the same conditions, they are similarly affected by technical noise, thus indicating that an improved SNR is obtained with the quantum-correlated probe. Furthermore, we confirmed that the SD of the peak field measurement for the classical case ($\sigma_{c}≃3.2$ μV) is compatible with the expected shot noise in the given acquisition conditions (bandwidth of B≃0.94 Hz at 2.1 kHz modulation frequency, transimpedance gain $G≃8.4\times 10^{6}$ ohms). The slight temporal compression visible in the quantum case is a consequence of the shorter (≃185 fs) duration of the squeezed pulses with respect to the coherent probe (≃245 fs), arising from the temporal compression occurring in the second harmonic generation process that provides the 515-nm pump pulse of the parametric amplification. By acquiring a statistical ensemble of points at each THz-probe delay, we assessed the signal SD over the whole 4-ps trace. As shown in Fig. 3B, the noise is uniformly distributed and does not depend on the delay, evidencing that the probe pulse is responsible for the primary source of noise in our acquisition conditions. The noise (SD of the signal) averaged of the whole trace is ${\bar{\sigma }}_{c}≃0.82$ μV for the classical measurement and ${\bar{\sigma }}_{q}≃0.43$ μV using two-mode squeezed pulses. As predicted by the measured NRF, the noise is reduced by nearly a factor of 2.

To properly assess the detection SNR (*42*), we sampled over 60 s the measured signal at the temporal coordinate of the THz peak, and we computed its SD, normalizing the peak signal to 1, for both probe configurations. The results for the classical and quantum probes are shown in Fig. 3 (C and D, respectively). The average normalized SDs in the two cases are ${\bar{\sigma }}_{r,c}≃0.051$ and ${\bar{\sigma }}_{r,q}≃0.025$, respectively, demonstrating that two-mode squeezing enables a twofold improvement of the SNR [defined as the ratio between the SD of the measured signal and its average value at the temporal coordinate of the THz peak field (*42*)] of the time-resolved electric field measurement with respect to what is possible with classical probes.

### Spectroscopy

**Fig. 4.**
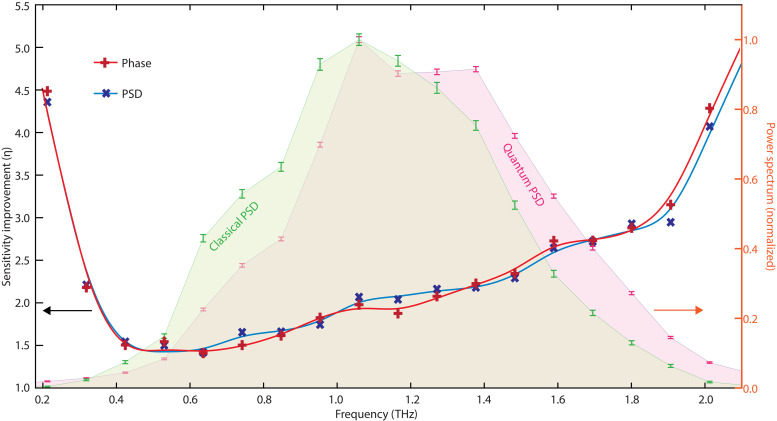
Spectral analysis. Normalized power spectral density (PSD, solid colors, right axis) for the classical (light green) and quantum (light purple) measurements, computed as explained in the text. Note the smaller error bars in the quantum case. The red and blue crosses show the values of the sensitivity improvement in the estimation of the spectral phase and power spectral density, respectively (left axis, see the main text for details). The solid blue and red curves are interpolants that serve as a guide for the eye.

From the recorded average and SD of the THz electric field at different THz-probe delays, it is possible to infer the expected noise reduction in the THz field spectral properties (amplitude and phase) via a Fourier transform. To this end, we numerically construct a series of *N* = 1000 temporal traces. Each temporal trace, for both the classical and quantum cases, is constructed by assigning an amplitude value to each delay coordinate. This amplitude is randomly selected from a normal distribution with mean being the average THz signal at the given delay and SD given by the measured average SD (which is different between the classical and the quantum measurement). From each temporal trace, a power spectrum and a spectral phase are obtained via Fourier transform $\left(\tilde{E}\left(\Omega \right)=F_{t}\left[ℇ\left(\tau \right)\right]\right)$. We then compute the average and the SD at each frequency point of the $\tilde{E}\left(\Omega \right)$ trace ensemble. In Fig. 4, we report the average power spectral density (PSD) $S\left(\Omega \right)={|\tilde{E}\left(\Omega \right)|}^{2}$ (shaded plots, values on the right axes) for the quantum (green) and classical (purple) cases. The slight difference in the power spectrum results from the difference in temporal traces, which, in turn, arises due to the different pulse durations of the quantum and classical probes. The uncertainty values on the power spectra values are shown with error bars. To clarify the quantum enhancement in the spectral domain, we computed the ratio between the relative errors of the classical measurement and that performed with the two-mode squeezed field at each frequency point

ηS(Ω)=σS,c(Ω)S¯c(Ω)σS,q(Ω)S¯q(Ω) (3)

where $\sigma_{S,c}$ and $\sigma_{S,q}$ are the SDs of the power spectral data at frequency Ω for the classical and quantum measurement, respectively, while ${\bar{S}}_{c}\left(\Omega \right)$ and ${\bar{S}}_{q}\left(\Omega \right)$ are the average classical and quantum PSD values at frequency Ω. This metric represents the possible increase in the sensitivity of power spectral measurements and is shown with the blue crosses (values on the left axis) in Fig. 4. $\eta_{S}>1$ over the whole spectrum, indicating an improvement in the sensitivity of the TDS performed with the two-mode squeezed radiation; furthermore, η > 2 over a large portion of the spectrum.

A similar metric can be defined for the spectral phase $\phi \left(\Omega \right)=\text{Arg}\left[\tilde{E}\left(\Omega \right)\right]$, such that

ηϕ(Ω)=σϕ,c(Ω)ϕ¯c(Ω)σϕ,q(Ω)ϕ¯q(Ω) (4)

where the same definitions used above for the PSD are now applied to the spectral phase ϕ. This metric measures the improvement in the measurement of the spectral phase that a detection system based on two-mode squeezed pulses could deliver over that performed with classical light, as shown in Fig. 4 (red crosses, left axis). This parameter, too, confirms a quantum-enhanced sensitivity in TDS.

## DISCUSSION

We have shown that two-mode squeezed light, a robust quantum metrology resource, can enhance the sensitivity of time-resolved field measurements, such as those underpinning TDS, providing a potential route toward sensitivities that are not achievable by classical means. Increasing the sensitivity of these time-resolved techniques not only will have a direct impact on TDS applications (*9*), but will also help shed light on the intriguing nonclassical effects arising at the subcycle level in nonlinear light-matter interactions (*26*, *43*–*45*). As a first demonstration of the concept, the results show potential for substantial improvement. Reduction of optical losses and improvement in detector quantum efficiency are technological challenges to be addressed, rather than fundamental physical limits. The use of squeezed light is more generally applicable, and the use of other measurement techniques (*26*, *46*) in combination with more complex nonclassical optical methodologies such as quantum-enhanced nonlinear interferometers is expected to yield even greater improvements (*47*–*49*). Nonetheless, we believe that having demonstrated a quantum advantage with probe power levels comparable to those routinely used in field-resolved spectroscopy will stimulate further development along this path toward quantum-enhanced field-resolved spectroscopy.

## MATERIALS AND METHODS

### Electro-optical sampling

The THz field is generated by OR of a 4.45-W, 245-fs-duration, p-polarized pump in a 1-mm-long, antireflection coated, (110)-cut GaP, oriented with the [001] crystallographic axis rotated at an angle θ_*z*_ ≃ −45° with respect to the *z*′ direction in the lab reference frame. This geometry generates an almost optimal THz signal (≃97% of the maximum amplitude) polarized at ≃108° with respect to *z*′ [see (*50*)]. To optimize the interaction, the THz radiation is initially sampled with a classical EOS based on a 300-μm-long, (110)-cut GaP crystal, with the [001] axis rotated at an angle of ≃39° with respect to the lab frame. The EOS is performed by blocking the idler in the setup shown in Fig. 1 (hence, using only the signal field) and using a QWP after the crystal and before the polarizer (instead of the HWP—shown in the figure). This resulted in an unbalanced signal from the photodiodes proportional to the THz electric field for probe (i.e., the signal) pulses polarized at 45° in the lab frame. This geometry provides near-optimal sensitivity, see e.g., (*51*). Once the spatiotemporal overlap between the THz and the signal pulse had been optimized, the idler path was opened and the QWP was replaced by an HWP. The idler pulses are several picoseconds delayed from the signal pulses so that they do not interact with the THz field and work as a reference. Note that the idler polarization is rotated by 90° with an HWP in its path and is, therefore, orthogonal to that of the signal. The detection HWP is tuned in such a way that signal and idler are almost perfectly separated by the Wollaston polarizer, and they produce a sub shot-noise differential signal on the balanced detector. A small, uncompensated component of the idler is projected to the signal path (and vice versa), which allows our detection scheme to be phase sensitive. Once the THz signal is allowed to interact with the signal in the GaP crystal, it slightly rotates the polarization of the latter, which is then measured by the balanced detector. Note that the bias differential signal due to the small HWP detuning from the θ = 22.5° angle is removed by the phase-locked detection scheme, which only extracts rotation components that are produced by the THz electric field.

### Sub shot-noise balanced detector

A low-noise balanced detector was designed and built based on a two-stage transimpedance amplifier. The first stage has a 10^6^-ohm transimpedance gain and employs the OPA827, high-precision, JFET-Input operational amplifier. A second stage, consisting of an OPA277, is a voltage buffer providing isolation between the circuit and the load impedance. InGaAs photodiodes (750 μm) were packaged by Bay Photonics (UK) in a TO-46 package, which includes three-stage thermoelectric cooling. As a result, the photodiodes could be cooled down to −50°C. By reverse biasing the devices, we were able to achieve a 50-fA Hz^−1/2^ input current noise within the measurement bandwidth and an external quantum efficiency of >94% (1030 nm).

### Two-mode squeezed light source

A 2-mm-long BBO crystal cut for type I parametric amplification (θ_*c*_ = 23.4) was chosen for the generation of the two-mode squeezed field. The length was limited by the splitting length between the 515-nm (185-fs) pump and the 1030-nm (245-fs) seed due to their different group velocities in the crystal. The parametric gain was adjusted to approximately 13.5 by pumping the crystal with 15-μJ pulses. This was determined to be the optimal operating point, striking a balance between achieving high gain for improved common-mode rejection ratio (CMRR) and mitigating the detectors’ limitations in effectively subtracting the spontaneous signal at high gain levels (*52*). To ensure lossless spatial separation of the signal and idler fields and to geometrically remove the optical pump, the interaction was noncollinear. A small angle of 2° between the pump and the seed directions was chosen to allow for pump-seed temporal overlap. To guarantee spatial overlap between the two input beams all along the crystal, the pump had a large diameter of ≃1 mm (1/e^2^) while that of the seed was ≃300 μm. The noncollinear angle was set in the plane orthogonal to the BBO optical axis to limit the spatial walk-off impact on the amplification process. Once the signal and idler (p-polarized) were generated, the idler was rotated by 90°. This enabled the spatial combination of the twin beams via a thin-film PBS, as shown in Fig. 1. As a result, both fields traverse the same media at identical locations, experiencing equivalent losses. To ascertain that the sub shot-noise differential signal measured by the balanced detector was indeed due to a nonclassical correlation, rather than an artifact of the measurement, we verified that the differential signal noise increases to the shot-noise level when mixing the signal and idler fields before splitting. To this end, the copropagating, cross-polarized signal and idler polarization were mixed by an antireflection-coated HWP placed before the Wollaston prism. By rotating the HWP, it was found that the NRF degraded, consistent with theoretical predictions.
